# The complete chloroplast genome sequence of the medicinal plant *Crotalaria albida*

**DOI:** 10.1080/23802359.2022.2080027

**Published:** 2022-06-07

**Authors:** Chunyang Jiang, Lipan Hu, Yingmei Wu, Lu Rui

**Affiliations:** College of Biology and Food Engineering, Chongqing Three Gorges University, Chongqing, P. R. China

**Keywords:** *Crotalaria albida*, complete chloroplast genome, Illumina sequencing, phylogenetic analysis

## Abstract

*Crotalaria albida* (*C. albida*) is a traditional Chinese medicinal plant that belongs to *Fabaceae* family. In this study, the complete chloroplast genome sequence of *C. albida* was sequenced. The genome is 152,743 bp in length and includes two inverted repeat regions of 25,535 bp. It was predicted to contain 127 genes in the chloroplast genome, among which 82 were protein-coding genes, 37 were tRNA genes, and 8 were rRNA genes. The maximum likelihood phylogenetic analysis based on 24 complete chloroplast genome sequences showed that *C. albida* was closely related to *Ormosia semicastrata*, *Ormosia emarginata*, and *Ormosia xylocarpa.*

*Crotalaria albida*, belonging to *Crotalaria* of *Fabaceae*, is an annual to short-lived perennial herb with multiple medicinal properties, mainly distributed in Anhui, Zhejiang, Fujian, Hunan, and Guizhou, China. The whole plant is used as a typical natural raw material for Chinese traditional medicine known as ‘HuangHuaDiDing’ (Huang et al. 1999), first described by Roth in 1821 (Roth, 1821). Pharmacological studies showed that its main chemical components have anti-tumor, antibacterial, and antiviral effects (Sun and Chou 2012). *Crotalaria albida* can also eliminate inflammation (Wei et al. 1999), relieve cough and fever, and treat carbuncle, swelling, and mastitis (Sivaramakrishna et al. 2021). Studies reporting on *C. albida* have mainly focused on investigating its morphological characteristics, chemical composition, and pharmacological activity (Hui et al. 1969). As an independent genetic unit, the chloroplast (cp) genome provides valuable information for species identification and phylogenetic analysis to conserve the species. Herein, we presented the complete chloroplast genome of *C. albida* to elucidate its genetic background and lay a foundation for further study and resource protection.

In this study, fresh leaves of *C. albida* were collected from Honghe County, Yunnan Province, China (102°06′58″E, 23°17′12″N). A specimen was deposited at the herbarium of the College of Biology and Food Engineering, Chongqing Three Gorges University. (https://www.sanxiau.edu.cn/smkx/index.htm, Nong Zhou and erhaizn@126.com) under the voucher number YHH15032. Total DNA was extracted from frozen leaves (snap-frozen in liquid nitrogen upon collection) according to the improved method using the CTAB extraction buffer reported by Kearse et al. (2012). The library was constructed with total DNA and sequenced using Illumina HiSeq 2500 platform (Novogene, Tianjin, China). To eliminate redundant data, the original reads were filtered by Trimmomatic v.0.32 software with default parameters (Bolger et al. 2014). Then, the clean reads were assembled into circular contigs by GetOrganelle (Jin et al. 2020) using cp genome annotation of *Crotalaria pallida* (GenBank accession number NC053562) as reference. Finally, the cpDNA was annotated by the Dual Organellar GenoMe Annotator GeSeq (Tillich et al. 2017) and CpGAVAS2 (Nguyen et al. 2015). The complete chloroplast genome was submitted to GenBank under accession number OL944396.

The circular chloroplast genome of *C. albida* is 152,743 bp in size with 36.6% GC content and comprises a large single copy (LSC) region (83,696 bp), a small single copy (SSC) region (17,977 bp) and two short inverted repeats (IRA and IRB) (25,535 bp each). The base composition of the circular chloroplast genome is A (31.3%), G (18.2%), C (18.4%), and T (32.1%). It was predicted that the cp genome contains 127 genes, including 82 protein-coding genes, 37 transfer RNA (tRNA) genes, and 8 ribosomal RNA (rRNA) genes. Moreover, 16 duplicated genes were found in the IR regions, which included 5 protein-coding genes, 7 tRNA genes, and 4 rRNA genes. The LSC and SSC regions contain 82 genes (60 protein-coding genes and 22 tRNA genes) and 13 genes (12 protein-coding genes, 1 tRNA gene), respectively.

To study the phylogenetic relationship of *C. albida* with other angiosperms, the complete chloroplast genome sequences of *Polygala japonica* and other 22 species belonging to *Fabaceae* were accessed from GenBank for analyses (Katoh and Standley 2013; Jin et al. 2020). Phylogenetic trees were constructed based on cp genome sequences using maximum-likelihood (ML) phylogenetic methods by MEGA7 performed with 1000 replicates (Kumar et al. 2018). As shown in Figure 1, *C**. albida* was clustered with *Ormosia semicastrata*, *Ormosia emarginata*, and *Ormosia xylocarpa.*

**Figure 1.**
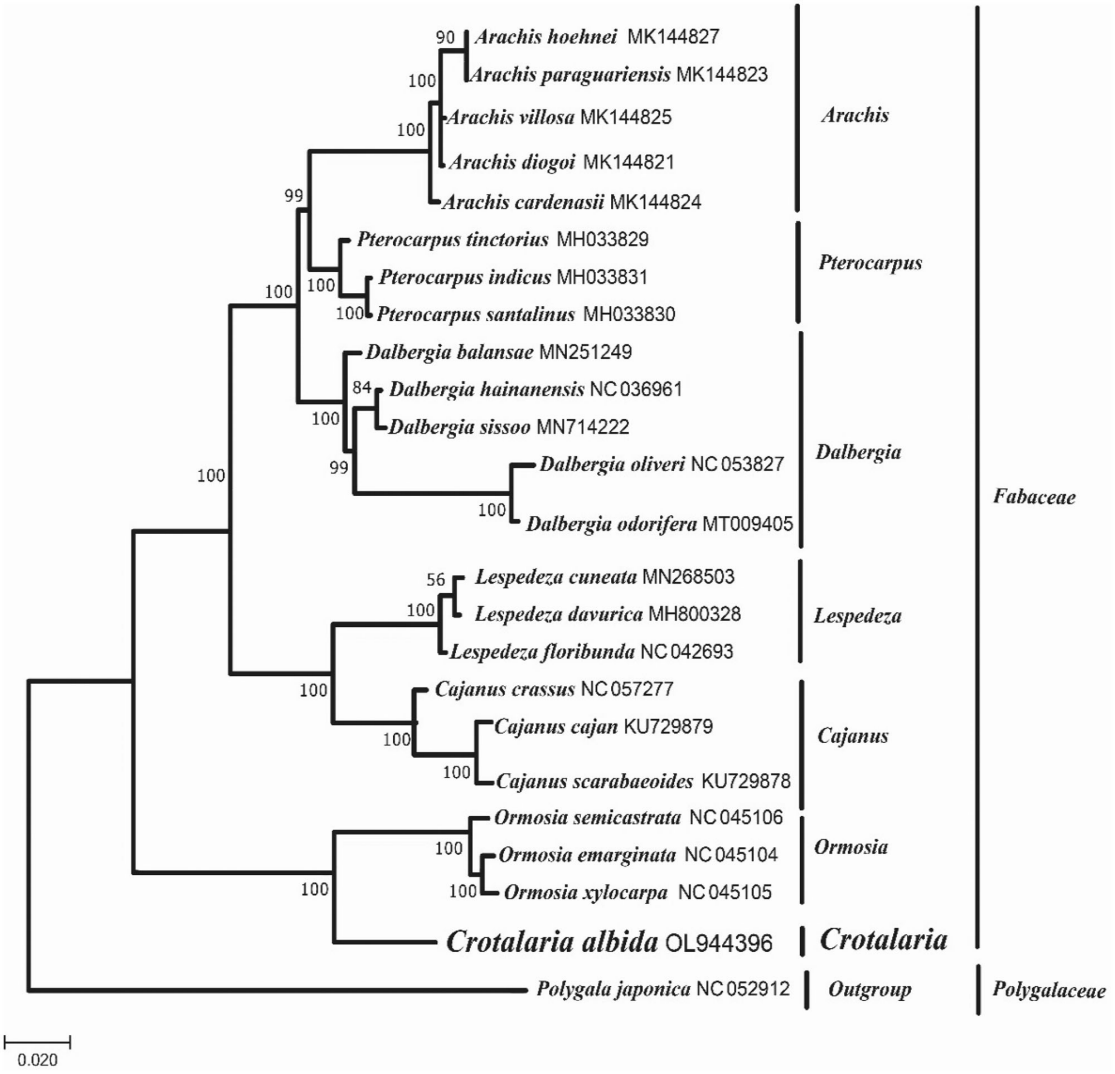
ML phylogenetic tree based on the complete chloroplast genome sequences of 24 species, including *C. albida*. The tree was rooted to *Polygala japonica* (NC052912). The cp genomes of the species used in this study were shown with its GenBank accession number followed. Bootstrap support values (1000 replicates) are shown next to the nodes. *Crotalaria albida* was in bold.

In this study, we reported the complete chloroplast genome of *C. albida*. These results provide valuable genomic information for developing molecular markers for molecular breeding and resource protection in the future.

## Data Availability

The data that support the findings of this study are openly available in GenBank of NCBI at https://www.ncbi.nlm.nih.gov, reference number OL944396. The associated BioProject, SRA, and Bio-Sample numbers are PRJNA814413, SRR18286790, and SAMN26550918, respectively.
